# Silibinin promotes healing in spinal cord injury through anti‐ferroptotic mechanisms

**DOI:** 10.1002/jsp2.1344

**Published:** 2024-07-01

**Authors:** Arman Vahabi, Anıl Murat Öztürk, Bünyamin Kılıçlı, Derviş Birim, Gizem Kaftan Öcal, Taner Dağcı, Güliz Armağan

**Affiliations:** ^1^ Department of Orthopaedics and Traumatology Ege University School of Medicine Izmir Turkey; ^2^ Faculty of Pharmacy, Department of Biochemistry Ege University Izmir Turkey; ^3^ Faculty of Pharmacy, Department of Biochemistry Afyonkarahisar Health Sciences University Afyonkarahisar Turkey; ^4^ Department of Physiology Ege University School of Medicine Izmir Turkey

**Keywords:** ferroptosis, iron chelation, lipid peroxidation, neuroregeneration

## Abstract

**Study Design:**

Pre‐clinical animal experiment.

**Objective:**

In this study, we investigated therapeutic effects of silibinin in a spinal cord injury (SCI) model. In SCI, loss of cells due to secondary damage mechanisms exceeds that caused by primary damage. Ferroptosis, which is iron‐dependent non‐apoptotic cell death, is shown to be influential in the pathogenesis of SCI.

**Methods:**

The study was conducted as an in vivo experiment using a total of 78 adult male/female Sprague Dawley rats. Groups were as follows: Sham, SCI, deferoxamine (DFO) treatment, and silibinin treatment. There were subgroups with follow‐up periods of 24 h, 72 h, and 6 weeks in all groups. Malondialdehyde (MDA), glutathione (GSH), and Fe^2+^ levels were measured by spectrophotometry. Glutathione peroxidase‐4 (GPX4), ferroportin (FPN), transferrin receptor (TfR1), and 4‐hydroxynonenal (4‐HNE)‐modified protein levels were assessed by Western blotting. Functional recovery was assessed using Basso–Beattie–Bresnahan test.

**Results:**

Silibinin achieved significant suppression in MDA and 4‐HNE levels compared to the SCI both in 72‐h and 6 weeks group (*p* < 0.05). GSH, GPX4, and FNP levels were found to be significantly higher in the silibinin 24 h, 72 h, and 6 weeks group compared to corresponding SCI groups (*p* < 0.05). Significant reduction in iron levels was observed in silibinin treated rats in 72 h and 6 weeks group (*p* < 0.05). Silibinin substantially suppressed TfR1 levels in 24 h and 72 h groups (*p* < 0.05). Significant difference among recovery capacities was observed as follows: Silibinin > DFO > SCI (*p* < 0.05).

**Conclusion:**

Impact of silibinin on iron metabolism and lipid peroxidation, both of which are features of ferroptosis, may contribute to therapeutic activity. Within this context, our findings posit silibinin as a potential therapeutic candidate possessing antiferroptotic properties in SCI model. Therapeutic agents capable of effectively and safely mitigating ferroptotic cell death hold the potential to be critical points of future clinical investigations.

## Graphical abstract

Ferroptosis, which is a lately described iron‐dependent non‐apoptotic cell death, is shown to be influential in the pathogenesis of spinal cord injury. Silibinin is a potential therapeutic candidate possessing antiferroptotic properties in spinal cord injury model.

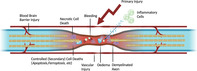

## 1 INTRODUCTION

Spinal cord injury (SCI) can be broadly classified into two phases: primary and secondary injury phases. Primary damage, which is the most critical factor influencing the extent of injury, results from the physical force at the time of the injury.^1^ Subsequent to the primary injury, secondary injury pathways including neuronal damage, inflammation, and hemolysis, are triggered.^2^ These pathways involve delayed and progressive damage mechanisms. As they come into play, the area of neuronal tissue damage expands, the degree of neurological impairment increases, and the likelihood of permanent damage or disability rises.^3^

**FIGURE 1:**
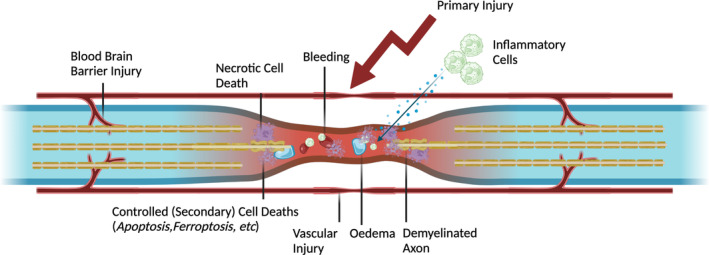
Spinal cord injury, physiopathological changes occurring in the acute injury phase.

It has been observed that the functional loss of cells due to secondary damage mechanisms exceeds what is caused by primary damage^4^ (Figure 1). In this context, a suppression of secondary damage by inhibiting secondary cell death mechanisms could lead to better functional recovery after SCIs. Ferroptosis, which is a lately described iron‐dependent non‐apoptotic cell death, is part of this secondary damage mechanisms, and it is shown to be influential in the pathogenesis of numerous diseases such as SCI. Recent literature demonstrates that following SCI, mechanisms leading to ferroptotic cell death are activated, and inhibiting ferroptosis yields positive effects on recovery.^5^, ^6^, ^7^ The molecular progression of ferroptosis involves the depletion of glutathione (GSH), simultaneous increase in intracellular iron, and the subsequent inactivation of glutathione peroxidase 4 (GPX4), which is responsible for reducing lipid peroxides. This cascade leads to an increase in lipid peroxidation and the presence of reactive oxygen species within the cell.^8^

Silymarin is a natural flavolignan derivative extracted from the fruits of *Silybum marianum*, commonly known as milk thistle. Silibinin, often referred to as silybin, is the primary active compound within silymarin, comprising 60%–70% of its composition. Numerous studies have explored the properties of the silibinin in various models, revealing its antioxidant, pro‐apoptotic, and anti‐inflammatory characteristics. These pharmacological effects have been corroborated by in vivo and clinical investigations, demonstrating its neuroprotective, hepatoprotective, anticancer, antidiabetic, cardioprotective, and immunomodulatory properties.^9^ Tsai et al. reported that silymarin and silibinin protect neuronal cells against oxidative stress and lipopolysaccharide stimulation.^10^ Also, silibinin has been shown to suppress lipid peroxidation and is able to bind iron; however, these mechanisms have not yet been associated with ferroptosis. In numerous studies exploring the effects of silibinin, considerable attention has been directed toward antioxidant pathways and apoptosis markers. However, investigations regarding the relationship between silibinin and ferroptosis remain limited and relatively recent in the literature. In an in vitro study investigating the protective mechanisms of silibinin against ethanol and acetaldehyde toxicity in liver cell lines, Song and colleagues demonstrated that silibinin mitigated alcoholic damage by targeting specific ferroptosis pathways in two distinct liver cell lines.^11^ Similarly, Yan et al. studied the impact of silibinin on HepG2 liver cell lines by inhibiting ACSL4, a key molecule in the ferroptosis pathway.^12^ Furthermore, a series of experiments conducted on hippocampal cell cultures unveiled that a combination of silibinin and taxifolin could exert neuroprotective effects through various pathways, including ferroptosis.^13^ Additionally, a study conducted on rat insulinoma INS‐1 cell cultures by Du et al. demonstrated that silibinin suppressed ferroptosis in insulinoma cell lines subjected to high glucose levels and palmitic acid treatment.^14^ But to date, there is no in vivo studies investigating the use of the silibinin as a therapeutic and antiferroptotic agent for SCI.

**FIGURE 2:**
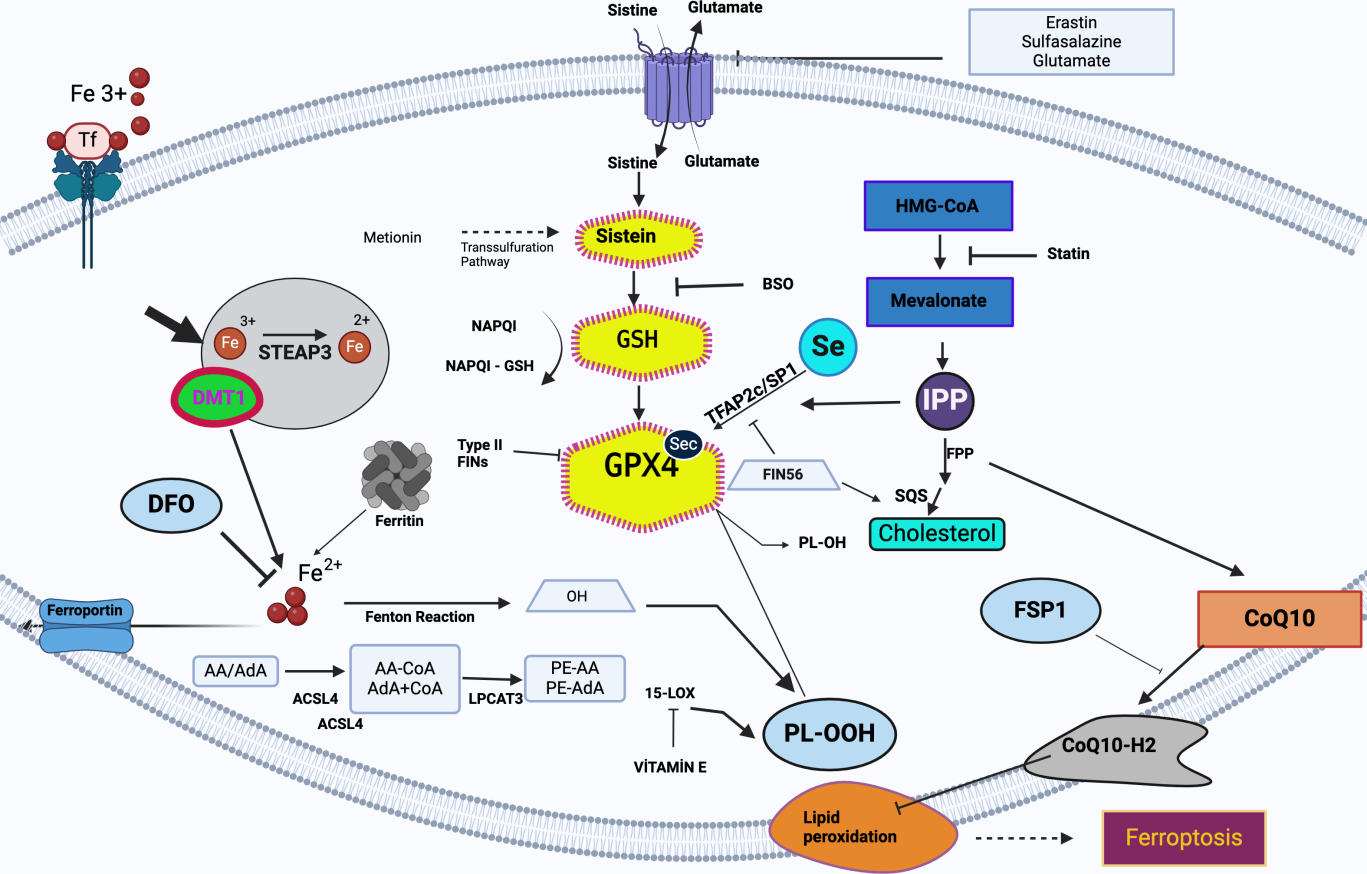
Pathways associated with ferroptosis and their interconnections.

In this study, we investigated the potential therapeutic effects of the silibinin when applied to a SCI model. We hypothesized that silibinin may have the capacity to mitigate secondary damage through inhibiting ferroptosis. This, in turn, leads to improved recovery and enhanced functional capacity. Our assessment primarily focused on two key biochemical pathways: iron accumulation and lipid peroxidation. To achieve this, we conducted measurements of iron and iron transporters (FPN and TfR1) levels, assessed lipid peroxidation markers (including 4‐HNE and MDA), and evaluated the levels of GPX4 and its main cofactor GSH which reduces complex hydroperoxides including phospholipid hydroperoxides (Figure 2). We have conducted comparative analysis of silibinin with deferoxamine (DFO), an antiferroptotic agent that has previously shown effective in a SCI model. This comparison was made to enable more objective interpretation of the results obtained rather than sole effort to prove silibinin's superiority over DFO regarding their antiferroptotic capacities. The administered doses were selected taking into account the safe and effective doses previously reported in the literature.

## 2 MATERIALS AND METHODS

The study was conducted as an in vivo experiment using a total of 78 adult (40 male/38 female) Sprague Dawley rats (10–12 weeks old, weighting 200–250 g) following the approval by the local ethics committee (26 May, 2021/2021‐046). All procedures were applied in accordance with Animals (Scientific Procedures) 1986 Regulations and its later amendments. Rats were housed in standard cages (one rat for each cage) with a 12‐h/12‐h light/dark cycle at 20–24°C with ad libidum feeding. The experimental groups were randomly divided into groups as follows: sham group (*n* = 15), SCI group (*n* = 21), SCI + deferoxamine group (SCI + DFO) (*n* = 21), and SCI+ silibinin group (SCI + Silibinin) (*n* = 21). These groups were further subdivided into three subgroups of seven animals (5 in sham subgroups) each with a specific follow‐up period of 24 h, 72 h, and 6 weeks. All subgroups were utilized for biochemical analyses. The 6‐week subgroups were also utilized for evaluating functional recovery.

### 2.1 Induction of SCI and drug treatments

On the day of the experiment, the animals were intraperitoneally anesthetized using ketamine hydrochloride (50 mg/kg; Ketalar, Parke‐Devis, Istanbul, Turkey) and xylazine hydrochloride (10 mg/kg; Rompun, Bayer, Istanbul, Turkey). Following a dorsal incision while in the prone position, the spinous processes and laminae were removed to expose the dorsal medulla spinalis. Aneurysmal clip application (50 g) was then performed at the T8–T10 levels, following the technique outlined by Rivlin and Tator, which entailed providing extradural compression for 60 s. In the sham group, the spinous processes and laminae were removed, but no damage was inflicted on the spinal cord.^15^, ^16^, ^17^, ^18^ Animals were observed daily, and bladder massage was performed two times a day until they re‐gain bladder control. Deferoxamine (100 mg/kg, Sigma‐Aldrich, D9533‐1G) dissolved in 0.9% NaCl was intraperitoneally injected following successful establishment of SCI.^19^, ^20^ Silibinin (200 mg/kg, Sigma‐Aldrich, S0417) was administered via gastric gavage daily.^21^, ^22^ In the groups sacrificed at the 24th hour, a single dose was administered. In other groups, drugs were administered to rats once a day until the third day. The animals in the sham group were intraperitoneally injected with vehicle (0.9% NaCl, 1 mL/kg). At the end of the treatment periods, animals were anesthetized with an overdose of ketamine. Spinal cords were dissected out on ice and 1 cm of cord segment centered at compression site were collected for biochemical analyses.

## 3 BIOCHEMICAL ANALYSES

### 3.1 Homogenization of spinal cord tissue

The spinal cord tissue samples of each group were washed with PBS and pooled together to increase the biological reproducibility of the results. At all groups, two separately pooled samples were analyzed in three technical replicates to confirm the results. Samples were lysed with an ice‐cold lysis buffer containing protease inhibitor cocktail (1:10 w/v), followed by centrifugation at 12000×*g* for 15 min at +4°C. The supernatant was used for further analyses. The concentrations of total proteins were quantified using the BCA kit (Thermo).

### 3.2 Determination of iron levels

The levels of ferrous (Fe^2+^) iron in spinal cord samples were determined using the iron assay kit (Sigma‐Aldrich, MAK025), according to the manufacturer's instructions. The optical density value was measured at 593 nm using a microplate reader (Varioskan Flash, Thermo Scientific, USA) and the iron levels (ng/μL) were determined by using the standard curve.

### 3.3 Determination of reduced glutathione levels

The reduced glutathione levels in spinal cord samples were determined using GSH Colorimetric Assay Kit (Elabscience, China, E‐BC‐K030) in accordance with the manufacturer's instructions. The GSH content was calculated by measuring the OD value at 420 nm. Results were expressed as miligrams GSH per grams of protein (mgGSH/g protein).

### 3.4 Measurement of MDA levels

The MDA levels in spinal cord samples were determined using a commercial TBARS Assay Kit (Cayman, USA) in accordance with the manufacturer's instructions. Briefly, the supernatant was mixed with SDS solution and color reagent. Then tubes were heated in a water bath for 1 h, followed by placing on ice bath for 10 min, and the optical density value was measured at 530 nm using a microplate reader (Varioskan Flash, Thermo Scientific, USA) and MDA levels in μM were determined by using the standard curve

### 3.5 Western blotting

To determine the levels of ferroportin (FPN), transferrin receptor (TfR1), 4‐hydroxynonenal (4‐HNE), and GPX4, 40 μg protein was loaded and seperated by electrophoresis, then transferred to PVDF membrane. After blocking, the membrane was incubated with primary antibodies (anti‐FPN rabbit polyclonal antibody [1:1000, Invitrogen]; anti‐TfR1 mouse monoclonal antibody [1:1000, Invitrogen]; anti‐4‐HNE mouse monoclonal antibody [1:1000, Invitrogen]; anti‐GPX4 mouse monoclonal antibody [1:500, Santa Cruz Biotechnology]) at +4°C overnight. Blots were washed twice with PBS‐T. Then, the membranes were incubated with horseradish peroxidase‐labeled anti‐rabbit or anti‐mouse IgG (1:5000) and developed by ECL using the SuperSignal West Pico Chemiluminescent Substrate (Thermo Scientific, Waltham, MA, USA). The blots were analyzed using the Fusion FX luminescence detector system (Vilber Lourmat, Marne‐La‐Vallée, France).

### 3.6 Behavioral assessment

For assessment of functional recovery, the Basso–Beattie–Bresnahan (BBB) behavioral test was employed to assess the functional outcomes following SCI.^23^ Functional assessments were employed for the groups undergoing a 6‐week follow‐up. We evaluated the Basso–Beattie–Bresnahan (BBB) locomotor scale at the end of the 1st, 2nd, 3rd, 4th, 5th, and 6th weeks to track changes in locomotor function over time.

### 3.7 Statistical analyses

Statistical analysis was carried out using the SPSS (Statistical Package for Social Sciences) v26 program. For categorical variables, frequency tables were generated, while continuous variables were described using mean values and standard deviations. Multiple group comparisons were conducted using one‐way ANOVA analysis of variance, and pairwise comparisons were performed with independent sample *t*‐tests. The averages of four groups at three distinct time points were compared using the repeated measures ANOVA method, with posthoc analysis conducted using the Bonferroni method. In all statistical tests, a two‐tailed alpha level (*α*) of 0.05 was set as the type 1 error rate.

## 4 RESULTS

### 4.1 Silibinin inhibits lipid peroxidation by decreasing MDA and 4‐HNE levels within 72 h after injury

**FIGURE 3:**
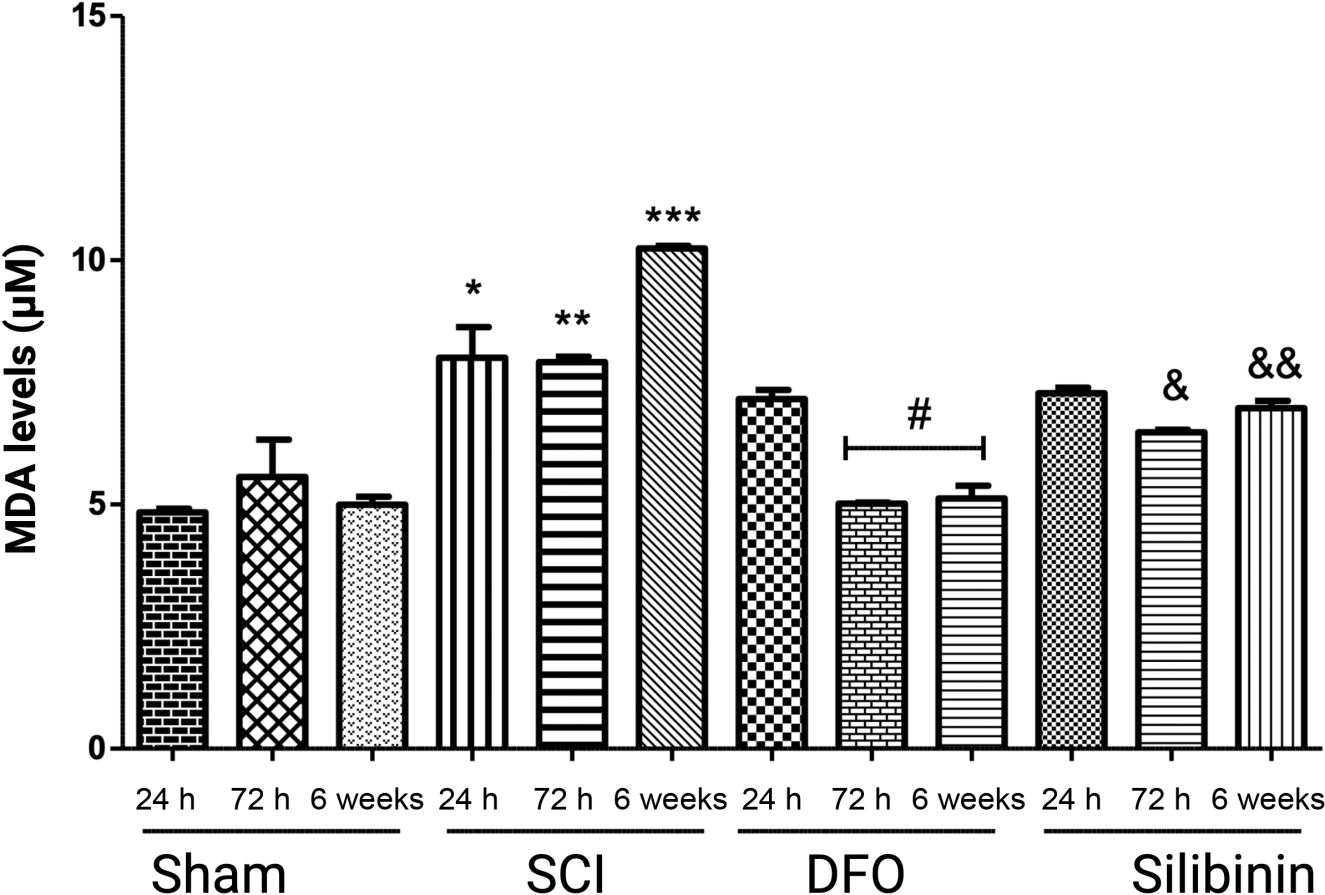
MDA (A) levels were significantly decreased in SCI + DFO and SCI + silibinin groups in the first 72 h after injury. *p < 0.05; SCI‐24 h group versus Sham‐24 h group; **p < 0.05; SCI‐72 h group versus Sham‐72 h group; ***p < 0.05; SCI‐6 week group versus sham‐6 week group; ^#^ *p* < 0.05; DFO‐72 h and 6 week group versus SCI‐all groups; ^&^ *p* < 0.05; silibinin‐72 h group versus SCI‐72 h group; ^&&^ *p* < 0.05; silibinin‐6 week group versus SCI‐6 week group. DFO, deferoxamine; MDA, malondialdehyde; SCI, spinal cord injury.

In the assessment of MDA levels at all three time points, elevated MDA levels were observed in the SCI group compared to the sham group (*p* < 0.05, *p* < 0.05, *p* < 0.05 respectively) (Figure 3). It was demonstrated that MDA significantly decreased in the silibinin‐treated group at 72 h (7.918 ± 0.11 to 6.482 ± 0.048, *p* < 0.05) and in 6‐week (10.243 ± 0.55 to 6.975 ± 0.148, *p* < 0.05). In the comparison of the silibinin groups with the DFO groups at 72 h and 6 weeks, MDA levels were significantly lower in the DFO groups (*p* < 0.05).

The repeated measures ANOVA assessing the impact of DFO and silibinin on MDA levels demonstrated a significant difference (*p* < 0.05, *p* < 0.05) when compared to the SCI group.

We detected higher 4‐HNE levels in SCI group compared to the sham group, confirming the activation of lipid peroxidation mechanisms in the injured spinal cord. DFO treatment significantly reduced 4‐HNE levels compared to the SCI at 24 h (1.719 ± 0.046 to 1.238 ± 0.038), 72 h (4.849 ± 0.110 to 0.753 ± 0.021) and 6‐week (5.294 ± 0.448 to 2.517 ± 0.1963). Although the 24‐h silibinin treatment could not effectively suppress 4‐HNE levels, 72‐h group (0.322 ± 0.027) achieved significant suppression of 4‐HNE‐modified protein levels (*p* < 0.05). Interestingly, in the comparison of the DFO 72‐h (0.753 ± 0.021) and silibinin 72‐h (0.322 ± 0.027) groups, it was evident that silibinin was more effective in suppressing lipid peroxidation, unlike the 24‐h groups (*p* < 0.05). Even when comparing the DFO 6‐week (2.517 ± 0.1963) and silibinin 6‐week (0.983 ± 0.097) groups, silibinin maintained its superiority in suppressing lipid peroxidation (*p* < 0.05). The repeated measures ANOVA assessing the impact of DFO and silibinin on 4‐HNE levels demonstrated a significant difference (*p* < 0.05, *p* < 0.05) when compared to the SCI group.

### 4.2 Glutathione levels and GSH‐dependent enzyme GPX4 expression are upregulated by silibinin in the first 24 h after SCI

**FIGURE 4:**
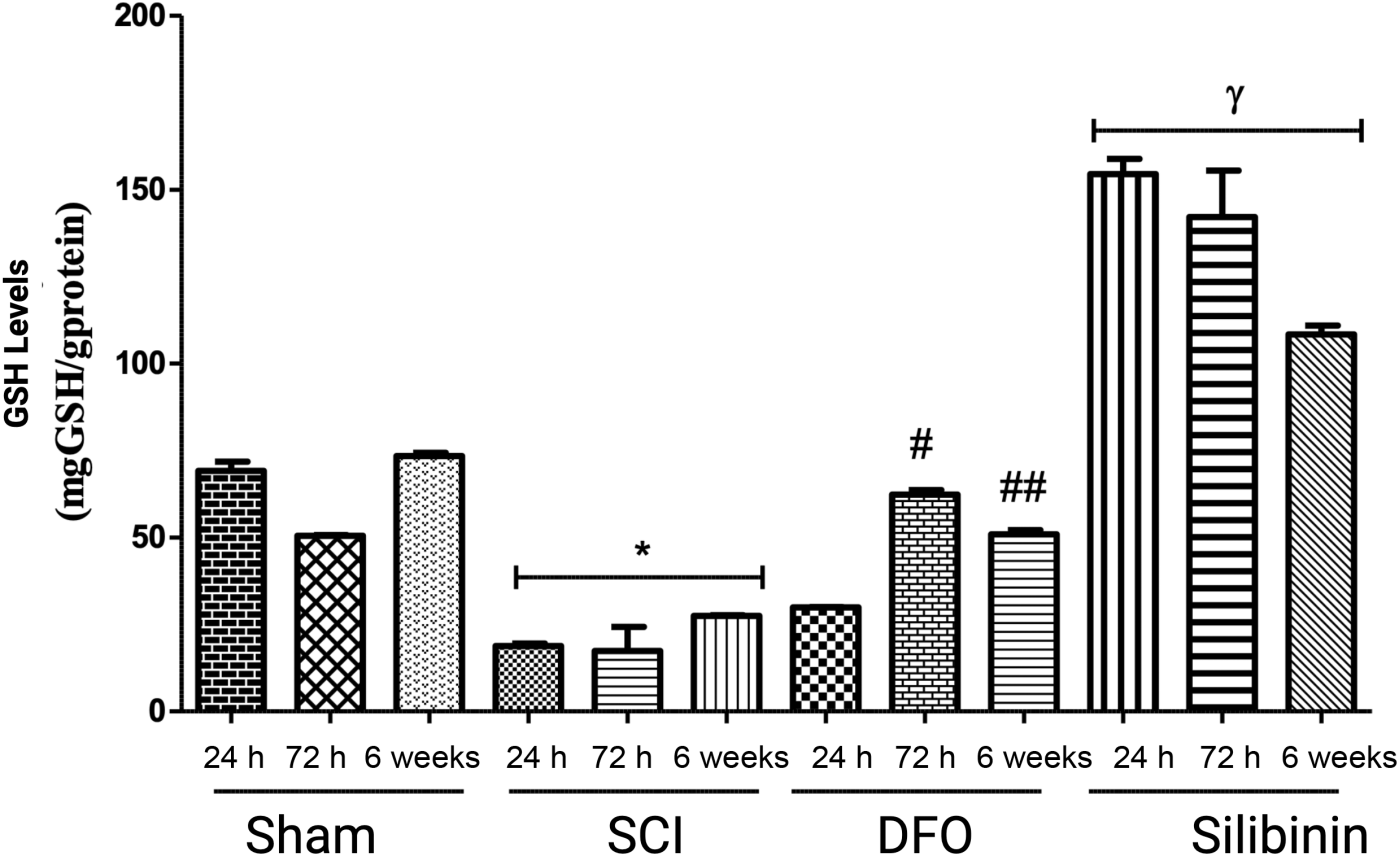
Alteration in GSH protein expression following DFO or silibinin treatments in SCI groups *p < 0.05; SCI‐all groups versus Sham‐all groups; ^#^ *p* < 0.05; DFO‐72 h group versus SCI‐72 h group; ^##^ *p* < 0.05; DFO‐6 week group versus SCI‐6 week group; ^γ^ *p* < 0.05; Silibinin‐ all groups versus SCI and DFO all groups. DFO, deferoxamine; SCI, spinal cord injury.

Regarding GSH levels, comparisons revealed that in the 24‐h, 72‐h, and 6‐week groups, GSH levels were notably lower in the SCI groups compared to the corresponding sham groups, signifying the effective induction of damage (*p* < 0.05, *p* < 0.05, *p* < 0.05, respectively). This reduction in GSH levels aligns with the expected response when ferroptosis is activated. In the comparative analysis of the silibinin and SCI groups, GSH levels were found to be significantly higher in the silibinin groups at all groups (*p* < 0.05). In the comparison between the DFO and silibinin groups, significantly higher levels of GSH were consistently observed in the silibinin‐treated groups (*p* < 0.05) (Figure 4). The repeated measures ANOVA assessing the impact of DFO and silibinin on GSH levels demonstrated a significant difference (*p* < 0.05, *p* < 0.05) when compared to the SCI group.

Regarding data on GPX4 levels, decreased GPX4 levels were observed in the SCI groups when compared to the sham groups, indicating the inactivation of antioxidant defense mechanisms in the injured spinal cord. Analyzing the 24 and 72‐h results, GPX4 levels significantly increased following DFO and silibinin treatments compared to the SCI (*p* < 0.05). Also, significantly higher GPX4 expression was observed in the DFO 72‐h, and 6‐week groups compared to the silibinin groups (*p* < 0.05). The repeated measures ANOVA assessing the impact of DFO and silibinin on GPX4 levels demonstrated a significant difference (*p* < 0.05, *p* < 0.05) when compared to the SCI group.

### 4.3 Silibinin protects spinal cord from ferroptosis by regulating the accumulation of iron after injury

**FIGURE 5:**
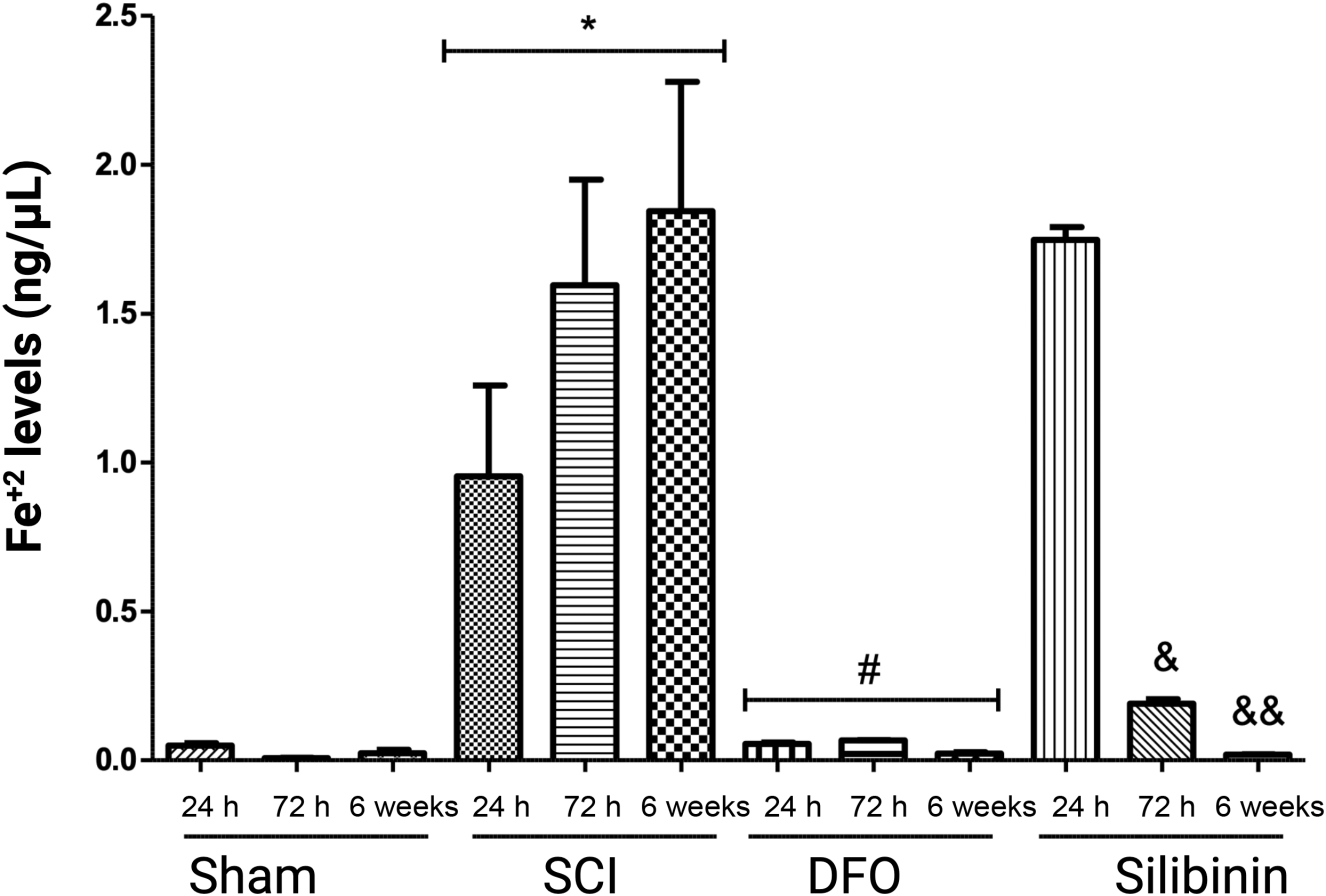
The changes in iron levels following DFO or silibinin treatments in SCI groups. *p < 0.05; SCI‐all groups versus sham‐all groups; ^#^ *p* < 0.05; DFO‐all groups versus SCI‐all groups; ^&^ *p* < 0.05; silibinin‐72 h group versus SCI‐72 h group^; &&^ *p* < 0.05; silibinin‐6 week group versus SCI‐6 week group. DFO, deferoxamine; SCI, spinal cord injury.

Fe^+2^ levels in the injured spinal cord increased across all three time periods when compared to the sham group (*p* = 0.052, *p* < 0.05, *p* < 0.05). Comparing SCI 72‐h group (1.595 ± 0.355) and the silibinin 72‐h group (0.190 ± 0.014) anticipated reduction in iron levels were achieved in silibinin treated rats (*p* < 0.05). The difference remained significant in the comparison of the SCI 6‐week group (1.844 ± 0.434) and the silibinin 6‐week group (0.019 ± 0.001) (*p* < 0.05). Comparing effects of DFO and silibinin on iron levels, DFO was more effective in suppressing iron levels in the 24‐h (*p* < 0.05) and 72‐h groups (*p* < 0.05). However, this superiority was not sustained in the 6‐week follow‐up period (*p* = 0.437) (Figure 5). The repeated measures ANOVA assessing the impact of DFO and silibinin on Fe^+2^ levels demonstrated a significant difference (*p* < 0.05, *p* < 0.05) when compared to the SCI group.

**FIGURE 6:**
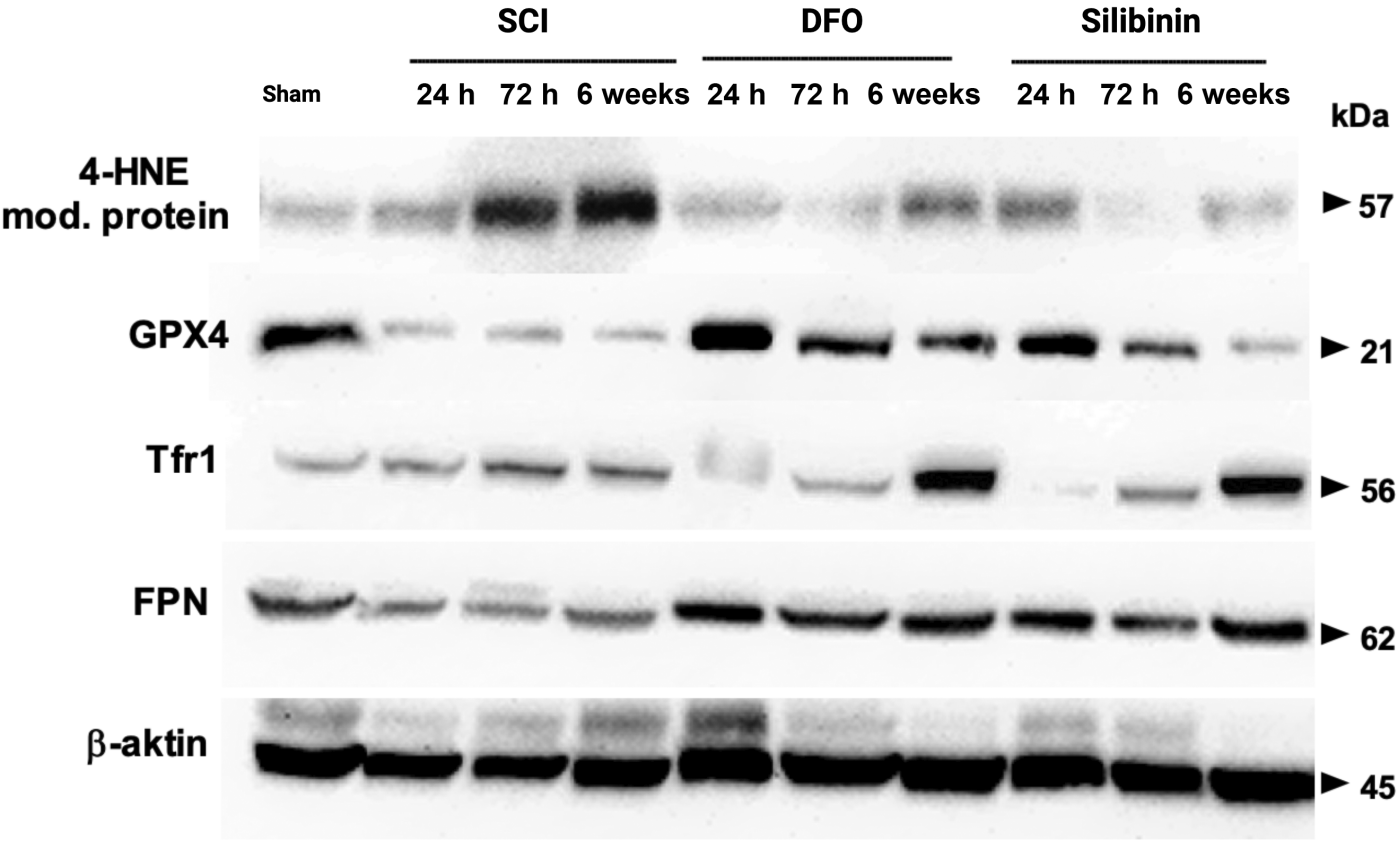
Western blot assessment on ferroportin, transferrin receptor‐1, glutathione peroxidase‐4 and 4‐hydroxynonenal.

Reduction in FPN levels was observed in the SCI group compared to the sham group, indicating the activation of ferroptosis mechanisms in the damaged spinal cord, coinciding with increased iron levels. Regarding 24‐h results, a significant increase in FPN levels was evident in the DFO (1.230 ± 0.013) and silibinin (1.108 ± 0.017) groups when compared to the SCI group (0.449 ± 0.009) (*p* < 0.05). Similar alterations were observed in 72 h and 6‐week groups of DFO and silibinin compared to the SCI groups. Furthermore, FPN levels were found to be notably higher in the DFO 72 h group (1.122 ± 0.012) than in the silibinin 72 h group (0.771 ± 0.014) (*p* < 0.05). In 6‐week groups, FPN levels were higher in the silibinin group (1.309 ± 0.044) than in the DFO group (1.247 ± 0.006); however, this difference did not reach statistical significance (*p* = 0.072) (Figure 6). The repeated measures ANOVA assessing the impact of DFO and silibinin on FPN levels demonstrated a significant difference (*p* < 0.05, *p* < 0.05) when compared to the SCI group.

Increased TfR1 levels were observed in the SCI group compared to the sham group, indicating an enhanced influx of Fe^+3^ into the cell due to the damage. In the 24‐h analysis, the DFO (0.758 ± 0.081) and silibinin (0.103 ± 0.024) treatments significantly decreased TfR1 levels compared to the SCI (1.304 ± 0.047) (*p* < 0.05). Notably, in the comparison of silibinin and DFO at 24 h, silibinin demonstrated more effective suppression of TfR1 levels (*p* < 0.05). Analyzing the 72‐h data, TfR1 levels were found to be significantly lower in the DFO group than in the silibinin group (*p* < 0.05). At the end of the 6‐week follow‐up period, TfR1 levels were significantly higher in the DFO (5.619 ± 0.252) and silibinin groups (5.158 ± 0.281) than in the SCI group (1.981 ± 0.004) (*p* < 0.05). It is worth noting that TfR1 levels in the silibinin 6 week group were lower than those in the DFO 6 week group, although not statistically significant (*p* = 0.1018). The repeated measures ANOVA assessing the impact of silibinin on TfR1 levels demonstrated a significant difference for silibinin (*p* < 0.05) when compared to the SCI group. There was no difference comparing DFO and SCI groups (*p* = 0.257).

### 4.4 Locomotor assessment

**FIGURE 7:**
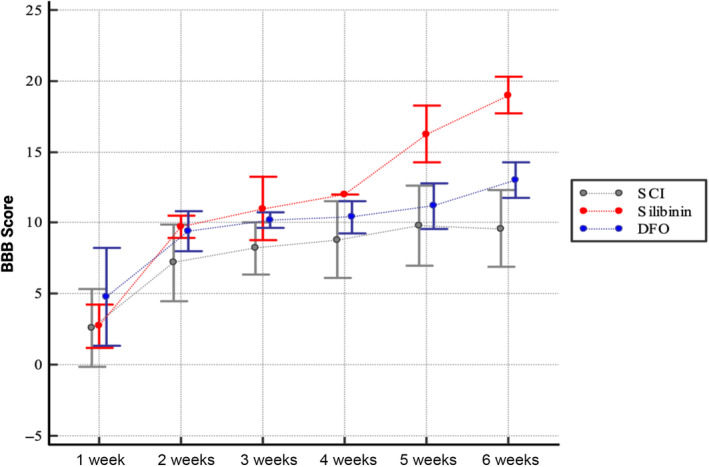
BBB score assesments in 1st, 2nd, 3rd, 4th, 5th and 6th weeks following DFO and silibinin treatments in SCI. BBB, Basso–Beattie–Bresnahan; DFO, deferoxamine; SCI, spinal cord injury.

For the sixth‐week evaluations, which represent the final time point on the temporal axis of locomotor activity level analysis, a statistically significant relationship among recovery capacities was observed as follows: Silibinin > DFO > SCI (Figure 7). Specifically, the positive effect of silibinin on recovery was highly significant when compared to SCI (*p* < 0.05), indicating a substantial enhancement in healing. Likewise, when comparing DFO to SCI, the positive effect of DFO on recovery was statistically significant (*p* < 0.05), signifying its role in promoting recovery. Furthermore, in the comparison between DFO and silibinin, silibinin was found to be superior (*p* < 0.05), indicating a more potent therapeutic effect over DFO.

## 5 DISCUSSION

SCI, a subject of intensive study, has yielded a limited number of therapeutic agents advancing to the clinical trial stage, despite numerous promising treatment candidates shown effective in preclinical animal models.^24^ Nevertheless, the current body of evidence collectively suggests that the expectation of achieving optimum recovery following SCI through monotherapeutic interventions targeting single pathways remains a distant prospect. Describing secondary damage pathways and discerning their interplay is of paramount significance.

Deferoxamine, the first iron chelator approved for clinical use, was shown to lead to enhanced functional recovery in rats by reducing free iron levels and mitigating lipid peroxidation pathways.^25^ In 2019, Yao et al. revealed mechanisms of deferoxamine's action in SCI, linking it to ferroptosis and characterizing DFO as an effective antiferroptotic agent in the context of SCI. In addition, Hao et al. reported that DFO treatment induced higher BBB scores, enhanced mitochondrial preservation, reduced iron levels, and increased expression of GPX4, xCT, and GSH as well as upregulation of ferroptosis‐related genes IREB2 and ACSF2 in SCI. This study represents a significant contribution, providing clear evidence that DFO can effectively serve as a treatment agent for spinal cord damage by modulating the ferroptosis pathway.^26^ Importantly, the findings from this study align closely with the data obtained in this study.

Because of several drawbacks of DFO such as short circulation time, toxicity concerns, limited delivery to brain, we aimed to evaluate silibinin, an active component of silymarin, as an antiferroptotic agent in SCI model, employing ferroptosis‐related parameters as indicators. *Silybum marianum*, a major source of silbinin, has been reported safe for humans at therapeutic doses and has few adverse reactions such as some gastrointestinal upsets.^27^ Previously, silibinin has been investigated in peripheral nerve injury models. A study focused on evaluating the outcomes of placing silibinin‐loaded chitosan nanoparticles in a rat model of nerve injury. The results indicated that loading the frameworks with silibinin nanoparticles led to improved histological and functional outcomes.^28^ Although the study did not investigate the biochemical pathways or mechanisms of action, the observed positive effects of silibinin on nerve healing suggest that similar action mechanisms and biochemical processes may be implicated in peripheral nerve injuries. This intriguing possibility warrants further exploration in future research endeavors. Yardım et al. investigated silymarin in both central and peripheral neurotoxicity models.^29^ In their study, the impact of silymarin was assessed through lipid peroxidation pathways, without specific reference to ferroptosis. Key biomarkers such as GSH, SOD, CAT, GPx, NF‐κB, TNF‐α, Nrf2, HO‐1, and Bcl‐2 were examined, revealing that silymarin exerts neuroprotective effects by modulating antioxidant defense mechanisms, thereby suppressing apoptosis and inflammation. Importantly, it is noteworthy that there is no distinct boundaries between controlled cell death pathways, particularly between apoptosis and ferroptosis. Nevertheless, the data obtained from our study hint at silibinin's influence on pathways beyond the lipid peroxidation stage, with the iron‐chelating property of silibinin potentially serving as a pivotal variable.

Regarding biochemical assessments of ferroptosis, we focused on two categories: lipid peroxidation and iron accumulation. Lipid peroxidation was estimated by assaying malondialdehyde and 4‐hydroxynonenal levels. MDA is anticipated to increase in processes associated with ferroptosis. While silibinin and DFO may not have a significant impact on reducing MDA levels in the initial 24 h following trauma, both effectively reduces MDA levels during the subsequent periods (72 h and 6 weeks) when ferroptotic cell death is ongoing. Findings indicate that the silibinin can significantly reduce MDA levels after SCI. However, it is worth noting that DFO appears to be more effective in reducing MDA levels, particularly starting from the 72nd hour onward. 4‐HNE serves as a marker of lipid peroxidation, with elevated levels indicating increased lipid peroxidation activity. Given that 4‐HNE is a highly reactive radical with a very short half‐life, we assessed the levels of 4‐HNE using 4‐HNE‐modified proteins. Evaluating the 4‐HNE levels for DFO, it is evident that its effectiveness diminishes over extended treatment periods. Conversely, in the silibinin group, we observe a consistent and significant decrease in 4‐HNE levels throughout the follow‐up period. In terms of reducing lipid peroxidation products, especially 4‐HNE, silibinin proves to be more effective than DFO.

Ferroptosis involves the depletion of GSH inside the cell, and the suppression of ferroptosis typically leads to increased GSH levels. The data regarding GSH levels collectively support the notion that silibinin exerts its antioxidant effects by involving GSH and suggests its potential to enhance the activity of GPX4, an enzyme crucial in the context of ferroptosis. GPX4 plays a key role in the repair of oxidative damage to lipids. A reduction in the levels of GPX4 is closely linked to increased ferroptotic activity. In simpler terms, the suppression of ferroptosis is anticipated to result in an elevation of GPX4 levels. Assessing the GPX4 data, it became apparent that both DFO and silibinin augmented GPX4 levels within the initial 24 h, with DFO demonstrating greater effectiveness in this regard. This trend persisted in the 72‐h and 6‐week groups, indicating the continued superiority of DFO over silibinin in enhancing GPX4 levels.

In scenarios where ferroptosis is inhibited through iron chelation, a significant reduction in iron levels is expected. To assess iron accumulation in spinal cord tissues, the levels of FPN, TfR1 and Fe^+2^ were measured. Although the inhibitory effects of silibinin on iron levels were not evident in the 24‐h groups, it became apparent in the 72‐h and 6‐week follow‐up groups. These findings indicate that the ferroptosis pathway is also instrumental in illustrating the effects of silibinin on the SCI model. TfR1 plays a role in importing Fe^+3^ into the cell, and its upregulation is characteristic of ferroptosis. Successful suppression of ferroptosis is associated with reduced TfR1 levels. The analysis of TfR1 levels suggests that both DFO and silibinin contributed to the regulation of intracellular iron levels within the first 24 h post‐injury, with silibinin showing greater efficacy. However, this advantage of silibinin over DFO was observed to reverse over time. It is noteworthy that the effects of silibinin on the TfR1 receptor may be short‐lived. Regarding the data from the 6‐week follow‐up, it becomes evident that both molecules exerted short‐term effects on TfR1 regulation. FPN is a crucial transmembrane protein located in the cell membrane, responsible for facilitating the export of free iron out of the cell. Inhibiting ferroportin can trigger ferroptosis by increasing intracellular iron levels. Hence, effective suppression of ferroptosis is expected to result in elevated FPN levels. Within the first 24 h, both DFO and silibinin contributed to the regulation of intracellular iron levels, albeit with DFO displaying greater efficacy. This trend persisted in the 72‐h groups. However, it is important to note that while this pattern reversed in the 6‐week groups, the data from the earlier stages hold more significance in interpreting ferroptosis dynamics.

In vivo study conducted by Tsai et al., the effects of silymarin and its active metabolite, silibinin, on oxidative stress and lipopolysaccharide stimulation in neuronal and glial cell cultures were thoroughly investigated. Their findings revealed that both silymarin and silibinin exhibited dose‐dependent inhibition of glial cell proliferation and provided protection against damage caused by reactive oxygen species. These effects were linked to the NFκB and PKC signaling pathways. Similar results were obtained in neuronal‐glial combined cell cultures, although silymarin demonstrated greater efficacy. In the in vivo stage of their study, intrathecal administration of silymarin was shown to have a positive impact on functional recovery.^10^ While it is acknowledged that natural compounds, particularly extracts derived from the entire product, may exhibit enhanced bioavailability owing to intermolecular interactions, this study specifically focused on silibinin only during the cell culture and did not extend to in vivo investigation. Notably, silibinin is recognized for its biological effects primarily through its active metabolite. Nonetheless, despite not establishing this correlation at the in vitro stage, the study still provided data consistent with our findings concerning the neuroprotective properties of silibinin. Our study further enhances the understanding of these effects by associating them with ferroptosis, thereby presenting this information in a more objective and applicable manner.

Our study had several drawbacks, including lack of histological examination. Our study focused primarily on protein levels, iron levels, and functional outcomes, necessitating careful planning of animal monitoring across three groups within our limitations. Similarly, evaluating ferroptosis‐related gene expressions would provide further objective data about the contribution of the ferroptotic pathway, which we failed to provide. Regarding interpretation the data presented, it is important to acknowledge that previously described secondary damage pathways (apoptosis, necroptosis, proptosis, autophagy, etc.) are intricately intertwined and interconnected processes and they are not independent pathways.

## 6 CONCLUSION

Silibinin demonstrates positive effects on healing in SCI model. Silibinin's impact on iron metabolism and lipid peroxidation pathways, both of which can be characterized as antiferroptotic properties, may contribute to this therapeutic activity. Ferroptotic cell death, along with its associated regulatory mechanisms, presents a multitude of unresolved queries, not only in the realm of SCI research but also in various injury mechanisms. Therapeutic agents capable of effectively and safely mitigating ferroptotic cell death hold the potential to be critical points of future clinical investigations.

## FUNDING INFORMATION

This study received financial support from the Ege University Scientific Research Project Coordination Office under grant numbers TGA‐2021‐23305 and TGA‐2021‐23 153.

## CONFLICT OF INTEREST STATEMENT

The authors declare no conflicts of interest.

## ETHICS STATEMENT

Ethical board approval for this study was obtained from Ege University Ethical Committee for Animal Experiments (26.05.2021/2021‐046). This study was conducted at Animal Laboratories of Ege University, Izmir, Turkey.
